# Bioprotective properties of seaweeds: *In vitro *evaluation of antioxidant activity and antimicrobial activity against food borne bacteria in relation to polyphenolic content

**DOI:** 10.1186/1472-6882-8-38

**Published:** 2008-07-10

**Authors:** Kasi Pandima Devi, Natarajan Suganthy, Periyanaina Kesika, Shanmugaiahthevar Karutha Pandian

**Affiliations:** 1 Department of Biotechnology, Alagappa University, Karaikudi- 630 003, Tamil Nadu, India

## Abstract

**Background:**

For many years chemical preservatives have been used in food, to act as either antimicrobials or antioxidants or both. In general, consumers regard additive-free foods as safer since preservatives can cause health hazards like asthma and cancer and are suspected to be mutagenic and neurotoxic. The present study was carried out to evaluate the antimicrobial and antioxidant activity of methanolic extracts of seaweeds, with a view to developing safer food preservatives.

**Methods:**

Ten edible seaweeds, which have wide pharmaceutical application, were collected from Central Marine Fisheries Research Institute, Tamil Nadu, India and evaluated for antioxidant and antimicrobial activity against food borne pathogens.

**Results:**

The results indicate that *Gelidiella acerosa *has the highest antioxidant activity while *Haligra sps *exhibited antibacterial activity against *Staphylococcus aureus *(MTCC 96).

**Conclusion:**

Quantitative analysis of the total phenolic content of the seaweeds indicated that *Gelidella acerosa *and *Haligra sps *have high phenolic contents, which correlated to their respective antioxidant and antimicrobial activity

## Background

Refrigerated, ready-to-eat products, especially dairy foods, have become increasingly popular in recent years because of their convenience. Many pathogenic organisms spoil such foods, reducing their shelf life and often leading to food poisoning. It has been estimated that as many as 30% people in industrialized countries suffer from a food poisoning every year [1,2]. In addition to microbial contamination, all packed and refrigerated food also undergoes gradual changes during storage, due to auto oxidation which releases reactive oxygen species (ROS) including free radicals like superoxide anion (O_2_^•-^) and hydroxyl radicals (OH^•^) and non-free radical species like singlet oxygen (^1^O_2_) and hydrogen peroxide (H_2_O_2_) [3,4] into the food. These ROS induce peroxidation of lipids (polyunsaturated fatty acids) generating secondary oxidants like heptanol and hexanal [5], which contributes to oxidative rancidity, deteriorating the flavor of the food [6]. These not only cause a loss in food quality but are also believed to be associated with carcinogenesis, mutagenesis, arthritis, diabetes, inflammation, cancer and genotoxicity [7-9]. To overcome these problems a wide range of synthetic antimicrobial agents (sodium benzoate, calcium benzoate, sorbate) and synthetic antioxidants (butylhydroquinone, propyl gallate, butylated hydroxy toluene (BHT), butylated hydroxy anisole (BHA) [10], have been used as food preservatives. However, these preservatives can cause liver damage and are suspected to be mutagenic and neurotoxic. Hence, most consumers prefer additive-free foods [11,12] or a safer approach like the utilization of more effective antioxidants and antimicrobials of natural origin [8,13]. Recently, various phytochemicals like polyphenols, which are widely distributed in plants, have been reported to act as free radical scavengers and antimicrobial agents [14,15]. Marine plants, like seaweeds, also contain high amounts of polyphenols. For example, high concentrations of polyphenols such as catechin, epicatechin, epigalloctechin gallate and gallic acid are reported in the seaweed *Halimada *(Chlorophyceae) [16]. Since many types of seaweed have still to be investigated, we were prompted to take up this study. The Gulf of Mannar is a Marine Biosphere Reserve situated along the east coast of India and Sri Lanka, an area of about 10,500 sq. km which has a luxuriant growth of about 680 species of seaweed belonging to the Rhodophyta, Pheaophyta and Chlorophyta, in both the inter-tidal and deep water regions. Seaweed constitutes a commercially important marine renewable resource. *Sargassum, Padina, Dictyota *and *Gracilaria sps*. Are used by common people as fertilizers, food additives and animal feed [17]. The sulphated polysaccharides of *Sargassum *act as a potent free radical scavenger and anticancer agent [18,19]. *Gelidella *and *Gracilaria sps *are widely used for the production of agar and for the treatment of gastrointestinal disorders [20]. The methanolic extract of brown seaweeds such as *Ecklonia cava *[21] and *Hizikia fusiformis *[22] exhibit potent antioxidant activity. Although seaweeds possess wide application in food and in the pharmaceutical industry, the antioxidant and antimicrobial activity of many types of seaweed in the South Indian coastal area are still unexplored. The main objective of the present study is to evaluate the antioxidant and antimicrobial activities of seaweeds obtained from the Thondi, South Coastal Area of Tamil Nadu, India.

## Methods

### Chemicals used

Nicotiamide adenine dinucleotide (NADH), thiobarbituric acid (TBA), nitroblue tetrazolium (NBT), DPPH (1, 1-diphenyl,2-picrylhydrazyl), 2,4,6-tripyridyl-S-triazine (TPTZ) were purchased from Sigma (Sigma-Aldrich GmbH, Sternheim, Germany). All other chemicals and solvents used were of the highest purity grade commercially available.

### Preparation of the seaweed extracts

Seaweeds were collected from Central Marine Fisheries Research Institute, Thondi, Tamil Nadu, and were identified by Dr. Nivedita Sahu, CSMCRI, India. The seaweeds used for study were *Gelidiella acerosa *(Rhodophyta), *Gracilaria edulis *(Rhodophyta), *Turbinaria conoides *(Phaeophyta), *Padina gymnospora *(Phaeophyta), *Chondrococcus hornemanni *(Rhodophyta), *Hypnea pannosa *(Rhodophyta), *Dictyota dichotoma *(Phaeophyta), *Jania rubens *(Rhodophyta), *Sargassum wightii *(Phaeophyta) and *Haligra *sps. The seaweeds were washed with water and alcohol, cut into small pieces and air-dried. One gm of dried sample was suspended in 10 ml of methanol for 72 h. The solution was filtered and evaporated to dryness under reduced pressure in a desiccator (Tarson Products Pvt Ltd, India). The dried powder was dissolved in distilled water containing less than 0.2% of methanol or Tween -20 (as solvents) and stored at -20°C until use [23].

### Antioxidant Activity

#### Free radical scavenging assay

The free radical scavenging activity of seaweed extracts was measured by DPPH according to Blois method [24]. 1 ml of DPPH^.^(0.1 mM) solution in methanol was added to 3 ml of methanolic extract of seaweed (100 μg/ml) in water, shaken vigorously, allowed to stand at RT for 30 min and the absorbance was measured at 517 nm in a UV- visible spectrophotometer (U-2800 model, Hitachi, Japan). A low absorbance of the reaction mixture indicated a high free radical scavenging activity. BHT (20 – 100 μg/ml) was used as positive control. The percent DPPH. scavenging effect was calculated as follows

$${\text{DPPH}}^{\cdot }\text{ Scavenging effect }(\%)=\frac{{\text{A}}_{\text{cont}}-{\text{A}}_{\text{test}}}{{\text{A}}_{\text{cont}}}\times 100$$

where A_cont _was the absorbance of the control reaction and *A*_test _was the absorbance in the presence of the sample seaweeds.

#### Hydroxyl Radical Scavenging Activity

The ability of seaweeds to scavenge OH^. ^was assessed using the classic deoxyribose degradation assay as described by Halliwell *et al *[25]. Briefly, 2.0 ml of the assay mixture containing EDTA (1 mM), FeCl_3_(10 mM), H_2_O_2 _(10 mM), deoxyribose (10 mM) and seaweed extract (100 μg/ml) was dissolved in distilled water with ascorbic acid (1 mM) in 50 mM phosphate The mixture was incubated at 37°C for 1 h and 1.0 ml of the incubated mixture was mixed with 1 ml of 10% TCA and 1 ml of 0.4% TBA (in glacial acetic acid, pH adjusted by NaOH) to develop the pink chromagen measured at 532 nm. BHT (20 to 100 μg/ml) was used as positive control. The hydroxyl radical scavenging activity of the extract is reported as % inhibition of deoxyribose degradation and was calculated as above.

#### Scavenging of Hydrogen Peroxide (H_2_O_2_)

The ability of the seaweeds to scavenge H_2_O_2 _was determined according to the method of Ruch *et al*., [26] with the slight modification of Gulcin *et al*., [27]. Briefly, 40 mM H_2_O_2 _was prepared in phosphate buffer (pH 7.4) and the H_2_O_2_concentration was determined spectrophotometrically by measuring the absorption with the extinction coefficient for H_2_O_2 _of 81 M^-1^cm^-1^. Extracts (100 μg/ml) in distilled water and ascorbic acid (20 – 100 μg/ml, positive control) were added to 0.6 ml of 40 mM H_2_O_2 _solution and the absorbance of H_2_O_2 _was determined at 230 nm after 10 min incubation against a blank solution containing phosphate buffer without H_2_O_2_. The percentage of scavenging of H_2_O_2 _was calculated as above

#### Nitric Oxide Radical (NO^.^) Scavenging Activity

NO^. ^generated from sodium nitroprusside in aqueous solution at physiological pH interacts with oxygen to produce nitrite ions, which were measured by the Griess reaction [28]. Briefly, 3 ml of the reaction mixture containing 10 mM sodium nitroprusside and the seaweed extract (100 μg/ml) in phosphate buffer were incubated at 25°C for 150 min. After incubation, 0.5 ml of the reaction mixture was mixed with 1 ml of sulfanilic acid reagent (0.33% in 20% glacial acetic acid) and allowed to stand for 5 min for complete diazotization. Then 1 ml of naphthyl ethylene diamine dihydrochloride (0.1%) was added and the solution mixed and allowed to stand for 30 min at 25°C. A pink colored chromophore is formed in diffused light. The absorbance of these solutions was measured at 540 nm against the corresponding blank solutions. BHT (50 – 250 μg/ml) was used as positive control. The NO^. ^scavenging activity of the seaweeds extract is reported as % inhibition and was calculated as above.

#### Total Reducing Power

Total reducing capacity of seaweeds was determined according to the method of Oyaizu [29]. The seaweed extract (100 μg/ml) in distilled water and 1% potassium ferricyanide were mixed with phosphate buffer (0.2 M, pH 6.6) and the mixture was incubated at 50°C for 20 min. 2.5 ml of 10% TCA was added to the reaction mixture which was centrifuged at 1000×g for 10 min. The upper layer of solution (2.5 ml) was mixed with distilled water (2.5 ml), FeCl_3 _(0.5 ml, 0.1%) and the absorbance was measure at 700 nm. Ascorbic acid (20 – 100 μg/ml) was used as positive control. The higher the absorbance of the reaction mixture the greater is the reducing power.

#### FRAP (Ferric reducing ability plasma) assay

The FRAP assay was performed according to the method of Benzie *et al *[30]. It depends on the ability of the sample to reduce the ferric tripyridyltriazine (Fe (III)-TPTZ) complex to ferrous tripyridyltriazine (Fe (II)-TPTZ) at low pH. Fe (II)-TPTZ has an intensive blue color which can be read at 593 nm. 1.5 ml of freshly prepared FRAP reagent (25 ml of 300 mM/L of acetate buffer pH 3.6, 2.5 ml of 10 mM/L 2,4,6 tripyridyl S triazine (TPTZ) in 40 mM/L of HCl, 20 mM/L of ferric chloride solution) were mixed with 50 μl of seaweed extract (100 μg/ml) in 150 μl of distilled water. The absorbance was monitored for 4 min (every 10 sec) at 593 nm. ΔA is proportional to the combined ferric reducing/antioxidant power (FRAP value) of the antioxidants in the sample. The results are expressed as mMol of FRAP/L and were estimated using aqueous FeSO_4 _.7H_2_O (200 – 1000 mM) as standard for calibration. The relative activity of the sample was compared with standard ascorbic acid (2–10 μg/ml).

#### Lipid peroxidation Assay

The lipid peroxidation level is measured as the thiobarbituric acid reactive substance (TBARS), based on the method of Yagi *et al *[31], with the limitation that the TBARS assay involves interference. Erythrocytes were hemolysed with an equal volume of ice-cold milliQ to yield 50% hemolysate, which was diluted to 1:20 with phosphate buffer containing 0.05 M dithioethreitol. The final volume of the reaction mixture was 1 ml, which consisted of 500 μl of the hemolysate, 300 μl of the buffer containing the plant extract (100 μg/ml) and 100 μl of 10 mM H_2_O_2 _to start the peroxidation. Samples were incubated at 37°C for 1 h, after which lipid peroxidation was measured using the reaction with thiobarbituric acid (TBA) to form thiobarbituric acid reactive substance (TBARS) a pink color chromogen read at 532 nm. BHT (20 – 100 μg/ml) was used as positive control. Lipid peroxidation is expressed as nM of malodialdehyde (nM)/gm of Hb, extrapolated from a standard curve prepared using known amounts of MDA.

#### Determination of Total phenolics

The total soluble phenolic compounds in the seaweeds were determined with the Folin-Ciocalteu reagent according to the method of Singleton *et al *[32] using gallic acid as standard. 100 μl of the sample (1 gm of dry sample in 10 ml of acetone) in duplicate was incubated with 1 ml of diluted Folin-Ciocalteu reagent (1:2 with water) at RT for 5 min. 1 ml of 7% Na_2_CO_3 _was added to the reaction mixture which was incubated at RT for 90 min. and the absorbance was read at 750 nm. The total phenolic content is expressed as gallic acid equivalent (GAE) in milligrams per gram of dry sample.

### Antimicrobial Assay

#### Bacterial strains

The microorganisms (food borne pathogens) used for assessing the antimicrobial activity of seaweeds were *Staphylococcusaureus *(MTCC 96), *Bacillus cereus *(MTCC 1272), *Vibrio vulnificans *(MTCC 1145), *Salmonella typhi *(MTCC 733), *Listeria monocytogens *(MTCC 657), *Enterococcus faecalis *(MTCC 2729) and *E.coli *0517; H7 (ATCC 43895),

#### Preparation of seaweed extract

Seaweed extract was filtered through a series of sterile, ethanol resistant cellulose acetate filters (0.2 μm, Advantec, Toyo Rashi Kaisha Ltd, Japan). The sterilized extract was stored in pre sterilized glass vials at -80°C until use.

#### Preparation of food samples

Ten grams of skimmed milk powder was mixed with 90 ml of distilled water and autoclaved at 121°C for 15 min prior to use to eliminate the contamination from organisms that may already be present in the food. The pH of the food samples ranged between 6 and 7.

#### Bacterial media

Brain Heart Infusion Agar (BHA), Brain Heart Infusion Broth (BHIB), Muller-Hinton Agar (MHA) and Muller-Hinton Broth (MHB), Nutrient Agar and Nutrient Broth were supplied by Hi media laboratories, India. Sodium benzoate was obtained from SRL chemicals Ltd, India. All media were prepared in deionised water and autoclaved at 121°C for 15 min prior to use.

#### Antibacterial Screening

The antibacterial activity of the seaweed extract was screened by the Disc diffusion method. All tests were performed in triplicate with different concentration of seaweed extracts. Sodium benzoate was used as the standard antimicrobial agent.

#### Agar – Disc Diffusion Method

Agar cultures of the test microorganisms were prepared as described by Mackeen *et al *[33]. Broth cultures were incubated overnight at 37°C. The concentration of the cultures was standardized by matching to the McFarland 0.5 turbidity standard using sterile saline to produce approximately 1.5 × 10^8 ^colony forming units (cfu) per ml. A suspension of the tested microorganisms was swabbed on the Muller Hinton agar medium. For screening, sterile 6 mm diameter filter paper disc were impregnated with different concentrations of plant extract (200 μg – 1 mg/ml). Discs were placed on MHA agar plates. Methanol or Tween-20 was used as negative control and sodium benzoate (200 μg) was used as positive control. Plates were incubated at 37°C for 24 h. Results were recorded by measuring the zones of growth inhibition surrounding the disc. Clear inhibition zones around the discs indicated the presence of antimicrobial activity. All data on antimicrobial activity are the average of triplicate analyses.

#### Determination of minimum bactericidal concentration (MBC)

The test substance was dispersed into an appropriate medium (MHB, reconstituted skimmed milk powder) and serial, two fold dilutions were made in the same medium to produce dilutions ranging from 1:10 to 1:640. 10 μl of standardized ON culture was added to each dilution. Following overnight incubation at 37°C, 50 μl from each dilution was spread on BHIA plates which were incubated overnight at 37°C. The procedure was performed in reconstituted skimmed milk powder. The minimum bactericidal concentration (MBC) was defined as the lowest concentration of plant extract that completely prevented microbial growth and was determined by visible inspection of the BHIA plates. MBC assays were carried out in duplicate.

### Statistical Analysis

All experiments were conducted in triplicate (n = 3) and one-way ANOVA (using SPSS 11.5 statistical software) was used to compare the mean values of each treatment. Significant differences between the means of parameters were determined by using the Duncan test (P < 0.05).

## Results

### Yield of seaweed Extracts

The % yield of the methanolic extract of *G. acerosa*, *G. edulis*, *T. conoides*, *P. gymnospora, C. hornemanni, H. pannosa*, *D. dichotoma*, *J. rubens*, *S. wightii *and *Haligra sps *were 4.5%, 1.5%, 5%, 3.5%, 1%, 4%, 11%, 2%, 5% and 5% respectively

### DPPH Radical Scavenging activity

**Figure 1:**
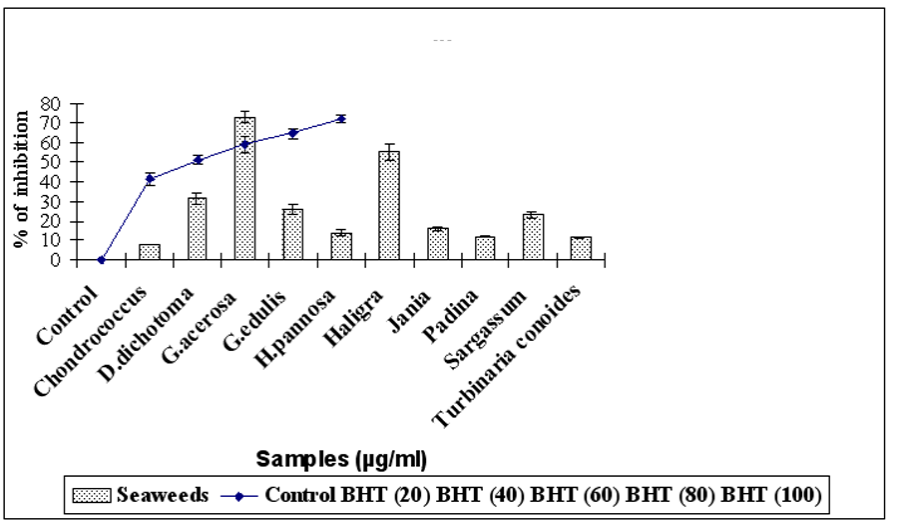
**Comparison of free radical scavenging activity of methanolic extract of seaweeds (100 μg/ml) with standard BHT (20–100 μg/ml).** Results are mean ± SD (n = 3).

The free radical scavenging activity of alcoholic extract of seaweed was assessed by the DPPH assay. Figure 1 illustrates a significant (P < 0.05) decrease in the concentration of DPPH radical due to scavenging ability of the seaweeds and the standard BHT. The results show that *G. acerosa *had the highest DPPH^• ^scavenging activity (72.5 ± 2.78%) among the seaweeds, significantly (P < 0.05) higher than that of the standard BHT (70 ± 2%). This indicates that *G. acerosa *as a good source of natural antioxidants.

### Lipid peroxidation

**Figure 2:**
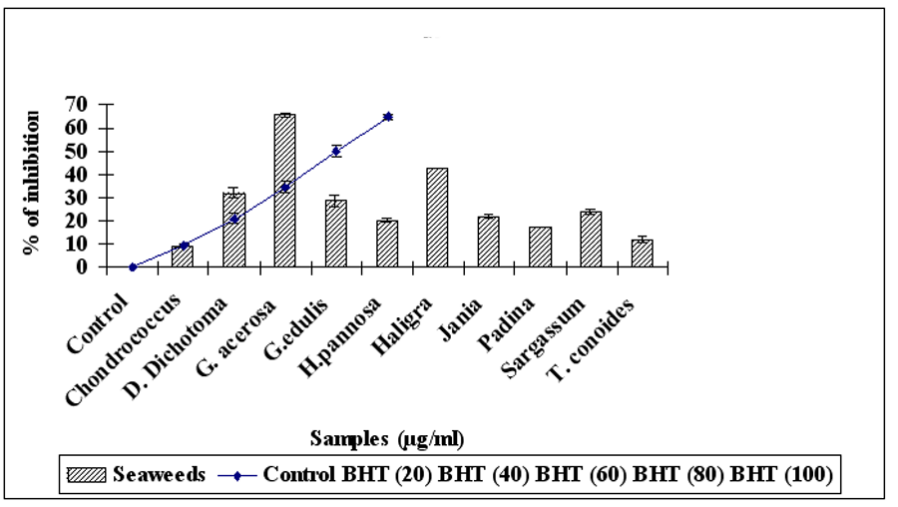
**Lipid peroxidation of methanolic fraction of seaweeds (100 μg/ml) in comparison with standard BHT (20 – 100 μg/ml).** Results are mean ± SD (n = 3).

The seaweeds not only exhibited excellent radical scavenging activity but also were potent in suppressing TBARS formation by H_2_O_2 _induced lipid peroxidation in RBC. Figure 2 show that *G. acerosa *(100 μg/ml) exhibited significant (P < 0.05) inhibition of 65.4 ± 0.9% of H_2_O_2 _induced lipid peroxidation when compared with other seaweeds. However it exhibited similar inhibition when compared with same dosage of BHT (65% ± 0.9%).

### Hydroxyl Radical scavenging activity

**Figure 3:**
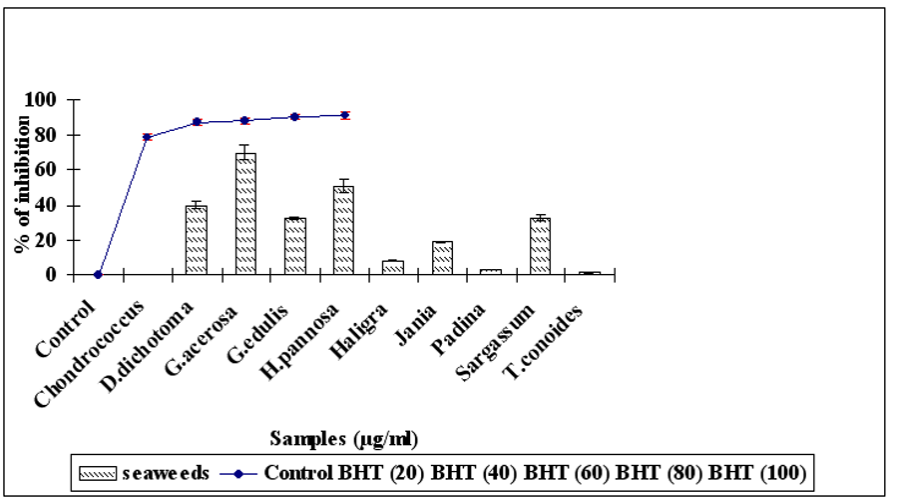
**Hydroxyl radical scavenging activity of Seaweeds (100 μg/ml) against positive control BHT (20–100 μg/ml).** Results are expressed as mean ± SD (n = 3).

The scavenging effect of OH^• ^was investigated using the Fenton reaction and the results areshown as an inhibition rate in Figure 3. *G. acerosa *exhibited the highest inhibition of about 70 ± 4.27% of the seaweeds but thisis lower than the standard BHT (100 μg/ml) whose % of inhibition is 89.15 ± 0.007% with a significance of P < 0.01.

### Nitric Oxide Radical scavenging Activity

**Figure 4:**
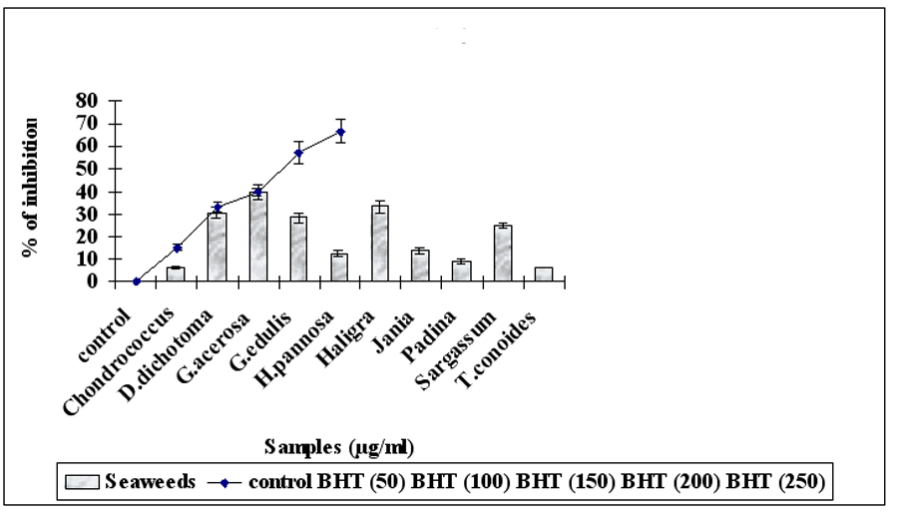
Scavenging effect of Seaweeds extract (100 μg/ml) and Standard BHT (50–250 μg/ml) on Nitric Oxide radical.

Suppression of NO^• ^release may be attributed to a direct NO^• ^scavenging effect as all the seaweed extracts decreased the amount of nitrite generated from the decomposition of sodium nitroprusside *in vitro *as shown in Figure 4. The results show that *Haligra sps *and *G. acerosa *(100 μg/ml) had scavenging activity of 39.8 ± 3.52 and 33.3 ± 1.7% respectively, similar to the standard BHT (33 ± 2.07%) with no significant differences.

### Scavenging of Hydrogen Peroxide

**Figure 5:**
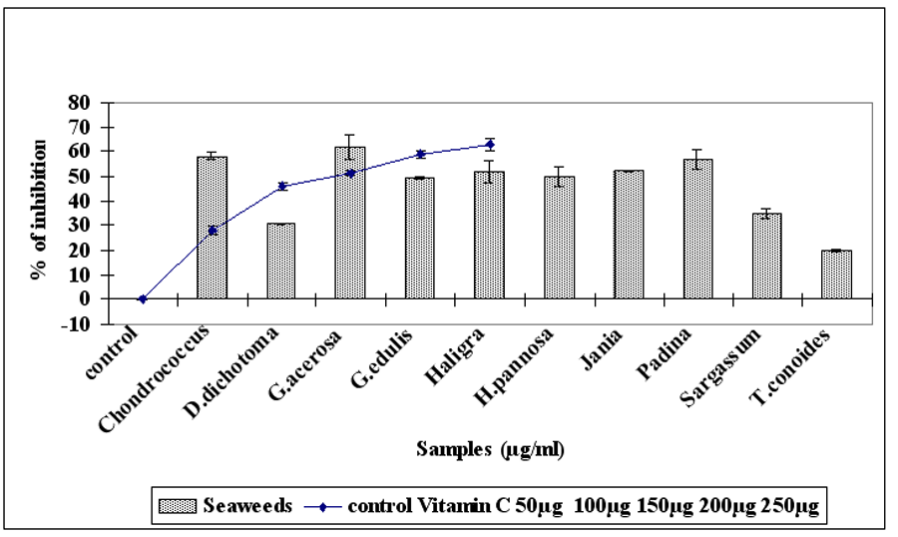
**Comparison of Percent Scavenging of Hydrogen peroxide radical by different seaweed extract (100 μg/ml) with same dosage of L-Ascorbic acid a positive control.** Results are expressed as mean ± SD (n = 3).

The ability of seaweeds to scavenge H_2_O_2 _was determined according to the method of Ruch and Gulcin *et al *[26,27]. Figure 5 indicates that *G. acerosa *(100 μg/ml) exhibits a maximum H_2_O_2_scavenging activity of 61.9 ± 1.27% which is significantly (P < 0.05) higher than the standard L-ascorbic acid whose scavenging effect is 51.7 ± 1.7%.

### Reducing power

**Figure 6:**
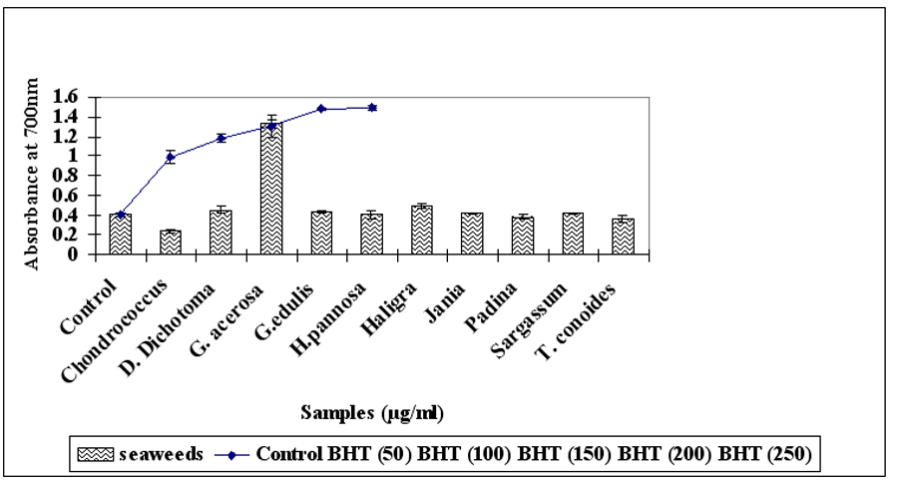
Reducing Ability of different seaweed extract and Standard BHT (50–250 μg/ml).

Figure 6 shows the reducing capacity of methanolic seaweed extract compared to standard BHT (50–250 μg/ml). *G. acerosa *(100 μg/ml) showed significantly (P < 0.01) higher reducing ability (absorbance 1.326 ± 0.042) when compared with the control (absorbance 0.402 ± 0.1) and the same dosage of the standard BHT (1.18 ± 0.043). Since the reducing capacity of a compound serves as a significant indicator of its potential antioxidant ability [34]*G. acerosa *can be considered as a potent source of natural antioxidants.

### Antioxidative power as measured by the FRAP assay

**Figure 7:**
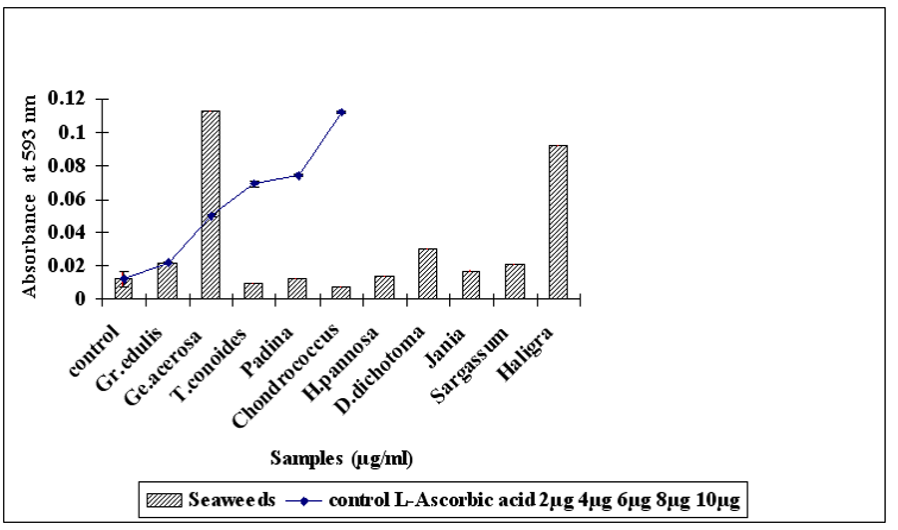
Total antioxidant power of methanolic extract of seaweeds(100 μg/ml) in comparison with L-Ascorbic acid (2–10 μg/ml) measured by FRAP assay.

The antioxidant power was measured by the FRAP method at λ = 593 nm which is based on comparison of the total amount of antioxidant with the reducing capacity of the samples. Figure 7 illustrates the total antioxidative power of the seaweed extracts (100 μg/ml) in comparison with vitamin C (2–10 μg/ml). FeSO_4 _was used as standard for calibration. The_. _FRAP value is expressed as mMol equivalent of Fe (II)/L. The results show that *G. acerosa *(100 μg/ml) exhibits significant (P < 0.05) ferric reducing capacity with an absorbance of 0.103 ± 0.005 which is equivalent to the absorbance of 800 mM/L of Fe (II) in FeSO_4 _in comparison with positive control vitamin C (10 μg/ml) with an absorbance of 0.112 ± 0.0005. When compared with the standard antioxidant L-ascorbic acid the antioxidative power was significantly less.

### Antimicrobial Activity

The antibacterial activities of ten seaweeds were assessed against seven food borne pathogens using a disc diffusion assay. Of the ten seaweeds screened *Haligra sps *(50 mg/ml) showed antibacterial activity against *S. aureus *(MTCC 96), a gram-positive bacteria, which causes food poisoning in humans. ZOI of *Haligra sps *was 16 ± 0.9 mm which is significantly (P < 0.05) higher than the standard antimicrobial agent sodium benzoate (200 mg/ml) whose ZOI was 14 ± 0.5 mm. The entire assay was carried out in triplicate.

The MBC assay performed in MHA and skimmed milk powder determined the lowest concentration of extract that produced a bactericidal effect. Table 1 shows the MBC result of *Haligra sps*. The highest MBC of *Haligra sps *extract against *S. aureus *was observed at a concentration of 7.5 mg/ml (1:20 dilution) in MHA and 5 mg/ml (1:40) in skimmed milk. Sodium benzoate, a standard antimicrobial agent, exhibited MBC at a concentration of 30 mg/ml (1:10) for both MHA and skimmed milk.

**Table 1:** Determination of MBC of *Haligra sps *extract against *Staphylococcus aureus *(MTCC 96)

**Sample**	**MHA**	**Milk**
*Haligra*	1:20	1:40
Sodium Benzoate	1:10	1:10

### Total Phenolic Compounds

**Figure 8:**
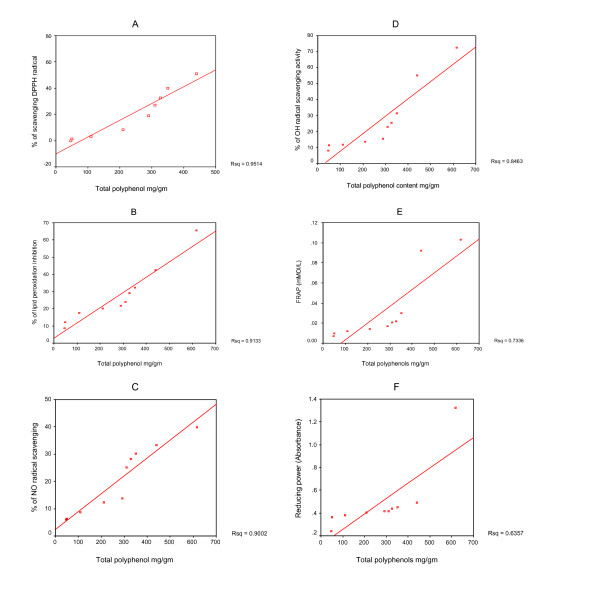
Correlation between the contents of total phenols in seaweeds and their antioxidant capacity as determined by Antioxidant Assay (AA) using DPPH method (A), Lipid peroxidation (B), Nitric oxide method (C), Hydroxyl radical method (D), FRAP method (E) and Reducing power (F).

The total phenolic content of the seaweed extracts was measured spectrophotometrically by the Folin-Ciocalteau method and the results are expressed as gallic acid equivalents (GAE). Table 2 shows that the total phenolic content of the *G. acerosa *(0.616 ± 0.0063 g/g) and *Haligra sps *(0.440 ± 0.0043 g/g) extracts was significantly higher than the other seaweeds. The correlation between the total polyphenol contents and the results of the determination of the antioxidant capacity were used to compare the sensitivity of the relevant tests as shown in Fig. 8(A, B, C, D, E, F). From the result it is clear that the correlation between the total polyphenolic content and antioxidant capacity, as determined by antioxidant assays using the DPPH^• ^scavenging assay, is the highest (R^2 ^= 0.9514) and with reducing power is the lowest (R^2 ^= 0.6357). Therefore the best method for determination the antioxidant capacity of seaweed is the DPPH method. We have already demonstrated that *G. acerosa *exhibits the greatest antioxidative activity and *Haligra sps *is the most effective antimicrobial agent when compared with the other seaweeds.

**Table 2:** Total Phenolic Content of Methanolic Extracts from Seaweeds^a^

**S.No:**	**Samples**	**Total phenolic content/gm (mg Gallic acid Equivalent/g)^b^**
1	Gellidela acerosa	616.3 ± 6.3
2	Gracilaria edulis	327 ± 13.5
3	Turbinaria conoides	50 ± 0.5
4	Padina	109.5 ± 1.9
5	Chondrococcus	47.5 ± 4.5
6	Hypnea pannosa	210.5 ± 2.1
7	Dictyota dichotoma	350 ± 3.5
8	Jania	290 ± 2.5
9	Sargassum	309.5 ± 3.9
10	Haligra	440.3 ± 4.3

^a ^The experiments were measured according to the Folin-Cioclateu method.

^b ^Each experiment was performed in triplicate and the results are mean ± SD.

## Discussion

Free radicals are highly reactive molecules with an unpaired electron and are produced by radiation or as by-products of metabolic processes. They initiate chain reactions which lead to disintegration of cell membranes and cell compounds, including lipids, proteins and nucleic acids. Besides damage to living cells, free radicals are the major cause of food deterioration through lipid oxidation, which ultimately affects the organoleptic properties and edibility of foods. Hence, intervention of an antioxidant may have a therapeutic effect and also maintain the freshness of food products. Antioxidant compounds scavenge free radicals such as peroxide, hydroperoxide or lipid peroxyl and thus reduce the level of oxidative stress and slow/prevent the development of complications associated with oxidative stress-related diseases [35] Many synthetic antioxidants have shown toxic and mutagenic effects, which have shifted attention towards naturally occurring antioxidants. A great number of naturally occurring substances like seaweeds have been recognized to have antioxidant abilities [36].

1,1-Diphenyl-2-picrylhydrazyl (DPPH) is a stable nitrogen centered free radical which can be effectively scavenged by antioxidants [37]. Hence it has been widely used for rapid evaluation of the antioxidant activity of plant and microbial extracts relative to other methods [38]. DPPH is also considered as a good kinetic model for peroxyl radicals [39]. The ability of seaweeds to scavenge DPPH radicals was determined by the decrease in its absorbance at 517 nm. The present investigation has shown that the extracts of all the seaweeds exhibited DPPH scavenging activity, the most effective being *G.acerosa *which exhibited significantly higher DPPH scavenging activity (72.5% inhibition) followed by *Haligra *species (55% inhibition) when compared with the highest concentration of the standard BHT (72% inhibition) (Fig 1). The result is indicative of the hydrogen donating ability of *G. acerosa*, since the effects of antioxidants on DPPH radical scavenging is thought to be due to their hydrogen donating ability [40]

In addition, the ability to scavenge the DPPH radicals is related to the inhibition of lipid peroxidation [41]. Hence the ability of the seaweeds to prevent lipid peroxidation was assessed in RBC by inducing lipid peroxidation with H_2_O_2_. During lipid peroxidation, low molecular weight end products, probably malonaldehyde, are formed by oxidation of polyunsaturated fatty acids and react with two molecules of thiobarbituric acid to give a pinkish red chromagen. As shown in Fig 2, at a concentration of 100 μg/ml, *G. acerosa *inhibited lipid peroxidation to 65.6% similar to the reference compound BHT which showed 64.9% inhibition.

OH^• ^has a short half-life and is the most reactive and damaging ROS. It causes oxidative damage to DNA, lipids and proteins [42]. The extract was examined for its ability to scavenge OH^• ^radicals generated by the Fenton reaction. When the seaweed extract and standard BHT (100 μg/ml) were added to the reaction mixture they removed hydroxyl radicals and prevented the degradation of 2-deoxyribose-2-ribose. *G. acerosa *exhibited the greatest scavenging effect of OH^• ^among the seaweeds but less than the standard BHT. OH^• ^is known to be capable of abstracting hydrogen atoms from membranes and they bring about peroxidic reactions of lipids [43]. It is thus anticipated that *G. acerosa *would show antioxidant effects against lipid peroxidation on biomembranes and would scavenge OH^• ^radicals at the stages of initiation and termination.

NO radicals play an important role in inducing inflammatory response and their toxicity multiplies only when they react with O_2_^•- ^radicals to form peroxynitrite, which damages biomolecules like proteins, lipids and nucleic acids [44]. Nitric oxide is generated when sodium nitroprusside reacts with oxygen to form nitrite. Seaweeds inhibit nitrite formation by competing with oxygen to react with nitric oxide directly. The methanolic extract of *G.acerosa and Haligra sp *at 100 μg/ml exhibited 39.8% and 33.3% inhibition which was comparable to the standard BHT, which exhibited 39.6% inhibition at 150 μg/ml. The present results suggest that *G.acerosa and Haligra sp *might be potent and novel therapeutic agents for scavenging of NO and the regulation of pathological conditions caused by excessive generation of NO and its oxidation product, peroxynitrite

Hydrogen peroxide is a weak oxidizing agent and can inactivate a few enzymes directly, usually by oxidation of essential thiol (-SH) groups. H_2_O_2_can cross cell membranes rapidly and once inside the cell it can probably react with Fe^2+ ^and possibly Cu^2+ ^to form hydroxyl radicals and this may be the origin of many of its toxic effects [45]. It is therefore advantageous for cells to control the amount of hydrogen peroxide that is allowed to accumulate. Figure 5 illustrates that the strongest anti- H_2_O_2 _activity was observed for *G.acerosa *at 100 μg/ml when compared with the standard BHT while *T conoides *appeared to be a very weak scavenger of hydrogen peroxide. The result suggest that *G acerosa *can be a better antioxidant for removing H_2_O_2 _and thus protecting food systems

The reducing ability of a compound greatly depends on the presence of reductones, which have exhibit antioxidative potential by breaking the free radical chain by donating a hydrogen atom [46]. For the measurement of reductive ability, we investigated the Fe^3+ ^to Fe^2+ ^transformation in the presence of an alcoholic extract using the method of Oyaizu et al [29]. Figure 6 illustrates that *G. acerosa *(100 μg/ml) showed higher absorbance when compared with the control and the same dosage as the standard BHT. The reducing capacity of *G. acerosa *is a significant indicator of its potential antioxidant activity. The results of the antioxidant assays indicate that *G. acerosa *is the best source of antioxidant compounds among the seaweeds investigated.

Apart from damage caused by free radicals, another common problem during food preservation is contamination by microbes. It not only affects the quality of food, but also causes serious health problems to those who consume the contaminated food. Of the ten seaweeds screened *Haligra sps *(50 mg/ml) showed antibacterial activity against *S. aureus *(MTCC 96) which was significantly (P < 0.05) higher than the standard antimicrobial agent sodium benzoate (200 mg/ml). The inhibition of growth of *S.aureus *by *Haligra sp *reveals that it can be used as an antibacterial agent against *S.aureus *which causes vomiting, diarrhoea, abdominal cramps and prostration and which also spoils raw meats, poultry, dairy products, salads, shrimp and ham [47].

The total phenolic content expressed as gallic acid equivalents was higher for the *G. acerosa *(0.616 ± 0.0063 g/g) and *Haligra sps *(0.440 ± 0.0043 g/g) extracts than for the other seaweeds. Plant phenolics in general are effective free radical scavengers and antioxidants. Phenolic compounds are commonly found in the edible brown, green and red seaweeds in which the antioxidative property has been correlated to their phenolic content [48-50]. Some authors claim that there is no correlation between the total phenolic content and the radical scavenging capacity [51], so it was very important to examine the correlation between the total phenolic contents and total antioxidant capacity of the studied seaweeds. The results of the present study reveal that there is a strong correlation between antioxidant activity and phenolic content. It is believed that the antioxidant properties of phenolics are a result of their ability to act as reducing agents, hydrogen donors, and free radical quenchers and phenolics can also act as metal chelators which prevent the catalytic function of metal in the process of initiating radicals [35]. It is possible that the antioxidant and antimicrobial activity of both the seaweed extract (*G. acerosa and Haligra*) can be the result of their high concentration of phenolic compounds.

## Conclusion

The present study elucidated for the first time the antioxidant property of ten seaweeds. This study suggested that, among the ten seaweeds, the *G. acerosa *extract possesses high antioxidant activity which might be helpful in preventing or slowing the progress of various oxidative stress related disorders. Moreover,*Haligra sps*. possesses potent antimicrobial activity against *S. aureus*, a food borne pathogen. Therefore it can be concluded that *G. acerosa *and *Haligra sps*, in appropriate combination, can act as an effective food preservative. There are few reports on the antioxidant capacity of seaweeds and the mechanism of seaweeds as antioxidative agents is still not fully understood. Hence ffurther research is underway to analyze and isolate the active compounds responsible for the antioxidant and antimicrobial activity from both the seaweeds.

## Competing interests

The authors declare that they have no competing interests.

## Authors' contributions

KPD and SKP conceived of the study, and participated in its design and coordination. PK carried out the assays. NS participated in the design of the study and performed the statistical analysis. All authors read and approved the final manuscript.

## Pre-publication history

The pre-publication history for this paper can be accessed here:


